# Synergistic Optimization of Reference Electrode and Solid Electrolyte for Bi/Bi_2_O_3_ Oxygen Sensors

**DOI:** 10.3390/ma19173771

**Published:** 2026-09-04

**Authors:** Guodong Liu, Shenghui Lu, Bo Qin, Zhangshun Ruan, Yuhui Wang, Lu Li, Xiaogang Fu, Naiqin Zhao

**Affiliations:** 1 School of Materials Science and Engineering, Tianjin University, Tianjin 300354, China; 2 China Institute of Atomic Energy, Beijing 102413, China

**Keywords:** lead-bismuth eutectic, Bi/Bi_2_O_3_ oxygen sensor, wide temperature range, synergistic optimization, long-term stability

## Abstract

The Bi/Bi_2_O_3_-type oxygen sensor is extensively employed for oxygen monitoring in liquid lead-bismuth eutectic (LBE)-cooled reactors, yet its low-temperature measurement accuracy remains a critical bottleneck limiting engineering deployment. This study aims to extend the lower operating temperature limit of the sensor through synergistic optimization of the reference electrode and solid electrolyte. The effects of the Bi/Bi_2_O_3_ mass ratio, filling amount, and yttria-partially stabilized zirconia (YPSZ) electrolyte wall thickness on sensor performance were systematically investigated over 300–600 °C. Electrochemical impedance spectroscopy and finite element simulations (COMSOL Multiphysics^®^ 6.3, COMSOL Inc., Stockholm, Sweden) were used to elucidate the underlying mechanisms. The results show that the optimized sensor with a Bi/Bi_2_O_3_ mass ratio of 95:5, a filling amount of 10 g, and a YPSZ wall thickness of 1.5 mm extended the stable operating limit from 350 °C to 300 °C, achieving a relative electromotive force error of 3.13% at 300 °C and maintaining over 4000 h of drift-free service. The improved low-temperature accuracy is attributed to the reduced oxygen ion migration activation energy (0.48 eV) and lower bulk impedance of the thick-walled YPSZ after high-temperature activation. These findings provide a material optimization strategy and theoretical basis for wide-temperature-range, long-lifetime oxygen sensing in lead-based reactors.

## 1. Introduction

Liquid lead-bismuth eutectic (LBE) has been selected as the primary coolant for lead-cooled fast reactors and accelerator-driven subcritical systems due to its excellent comprehensive properties [1,2,3]. However, controlling the dissolved oxygen concentration in LBE is a core technical challenge for ensuring the compatibility between structural materials and the coolant: an excessively low oxygen concentration leads to the dissolution corrosion of stainless steel components, while an excessively high concentration results in the formation of solid PbO particles that can cause flow channel blockages [4,5,6,7]. Therefore, the dissolved oxygen concentration must be precisely controlled in real time within a narrow “oxygen window” (typically 10^−8^–10^−6^ wt.%) [7,8]. Research on the corrosion of structural materials in LBE [9,10] has demonstrated that oxygen concentration control represents the most promising engineering approach for achieving long-term protection of structural materials. Furthermore, progress in the compatibility evaluation of stainless steels for lead-cooled fast reactors in liquid lead-bismuth environments [11] has provided important references for structural material selection and the determination of oxygen control boundaries.

Electrochemical oxygen sensors based on zirconia solid electrolytes represent the mainstream approach for oxygen measurement in LBE [12,13], among which the Bi/Bi_2_O_3_ type has become the preferred choice because Bi is a natural component of LBE and the sensor can be fully solid-state sealed [8,14]. Shooshtari and Salehi [13] showed that designing composite materials can create beneficial synergies that overcome the performance limits of single materials—a concept that also applies to the multi-parameter optimization of oxygen sensors. A comprehensive review [8] systematically summarized the progress of oxygen measurement and control technologies for LBE in the context of material corrosion protection, further confirming the outstanding advantages of the Bi/Bi_2_O_3_ system in terms of sealing reliability and LBE compatibility. However, extensive research has revealed two common bottlenecks: first, a significant degradation in measurement accuracy at low temperatures (<350 °C)—operating data spanning tens of thousands of hours from KIT in Germany indicated that Bi/Bi_2_O_3_ sensors suffer from pronounced signal drift and electrolyte cracking during frequent start-stop cycles [15], and systematic comparisons by SCK-CEN in Belgium [16,17] and ENEA in Italy [18] further confirmed that the reliable lower operating limit is approximately 350 °C, with experiments at the China Institute of Atomic Energy also showing significant deviations below 375 °C [19]; second, the failure mechanisms during long-term service remain unclear, with most existing validation studies limited to hundreds of hours, making it difficult to support engineering applications.

In recent years, new progress has been made in the field of oxygen sensors. Tu et al. [20] systematically analyzed the influence mechanism of solid electrolyte material selection on EMF characteristics, involving the volume expansion effect of the Bi solidification phase transformation, providing a theoretical reference for YPSZ engineering selection. Geng et al. [21] comparatively evaluated the performance of Cu/Cu_2_O- and Ni/NiO-based potentiometric oxygen sensors in saturated oxygen LBE, revealing the decisive influence of the reference electrode composition on measurement accuracy. Recent studies have further confirmed the effectiveness of electrochemical oxygen pumps in LBE oxygen concentration control, with control efficiency significantly improving as temperature increases [22]. Shooshtari [23] further showed that noble metal nanoparticle decoration can significantly increase the density of active sites and promote charge transfer, thereby enhancing sensor sensitivity. Domestically, Wang et al. [24] verified the short-term accuracy of Bi/Bi_2_O_3_ sensors, Zhu et al. [25] confirmed the feasibility of Cu/Cu_2_O reference electrodes, and Li [26] explored performance optimization strategies for low-temperature oxygen sensors. In addition, the thermal stress damage to YPSZ ceramic tubes caused by the approximately 3.3% volume expansion of Bi during solidification at 271 °C [20,27] and the intrinsic influence of YPSZ wall thickness on oxygen ion conduction [27,28] still lack systematic experimental and simulation analysis.

To address the above issues, this paper focuses on the synergistic optimization of the reference electrode and solid electrolyte of Bi/Bi_2_O_3_ oxygen sensors. Unlike previous studies that typically optimized only a single component (e.g., reference electrode composition or electrolyte material alone), this work establishes a multi-parameter synergistic optimization strategy—simultaneously tuning the Bi/Bi_2_O_3_ mass ratio, filling amount, and YPSZ wall thickness—to extend the low-temperature operational limit while maintaining high accuracy and long-term stability. Using a self-designed welded sealing sensor, this study systematically investigated the regulatory effects of the Bi/Bi_2_O_3_ mass ratio, filling amount, and YPSZ wall thickness on the low-temperature measurement accuracy, dynamic response, and long-term stability of the sensor. Electrochemical impedance spectroscopy (EIS) was employed to elucidate the intrinsic influence of wall thickness on oxygen ion conduction, and COMSOL Multiphysics simulations were combined to reveal the mechanical mechanism of electrolyte cracking induced by the Bi solidification phase transformation. This study aims to provide a material strategy and theoretical basis for wide-temperature-range and long-lifetime oxygen monitoring in lead-based reactors.

## 2. Materials and Methods

### 2.1. Sensor Design and Assembly

The oxygen sensor consisted of a YPSZ solid electrolyte tube, a Bi/Bi_2_O_3_ reference electrode, a Mo lead wire (purity 99.95%, diameter 1.0 mm, Suzhou Chuanmao Metal Materials Co., Ltd., Suzhou, China), and a 316L stainless steel shell(Suzhou Chuanmao Metal Materials Co., Ltd., Suzhou, China). Referring to the sealing design concept of oxygen probes for liquid lead-bismuth alloys [29], this study adopted a welded sealing process to achieve the ceramic–metal connection, ensuring the hermetic reliability of the sensor during long-term service. The YPSZ ceramic tube had a Y_2_O_3_ doping content of approximately 5 mol%, an outer diameter of 10 mm, and inner diameters of 7.0 mm (wall thickness 1.5 mm) and 7.8 mm (wall thickness 1.1 mm), with one end closed.

Two Bi/Bi_2_O_3_ mass ratios, 95:5 and 90:10, were selected for investigation. This selection was based on established engineering practice: to prevent the metal from being completely oxidized and to account for the inevitable oxide film on fine metal powder, as well as to ensure sufficient electronic conductivity, the proportion of metallic powder in metal/metal oxide reference electrodes is generally taken as 90% or above [24]. Thus, 90:10 represents the practical lower limit, while 95:5 was chosen as a representative Bi-rich formulation to explore the benefits of further increasing the Bi content for low-temperature performance. Bi powder (purity 99.99%, 200 mesh, Aladdin Biochemical Technology Co., Ltd., Shanghai, China) and Bi_2_O_3_ powder (purity 99.0%, Aladdin Biochemical Technology Co., Ltd., Shanghai, China) were ground and mixed.

Three filling amounts—5.7 g, 10 g, and 15 g—were investigated. The 10 g amount was initially selected as the baseline, as it produces a molten Bi column height of approximately 10 mm in the YPSZ tube, ensuring sufficient effective contact area between the reference electrode and the electrolyte while avoiding an excessive oxygen potential gradient. The 5.7 g amount (molten height ~6 mm) was chosen to simulate the condition of insufficient active material, while the 15 g amount (molten height ~15 mm) was selected to examine the effect of an excessively thick powder layer on oxygen potential equilibrium and activation behavior. The ceramic–metal connection was achieved using a brazed sealing head with a built-in alumina insulation structure to achieve electrical isolation.

### 2.2. Experimental Setup and Test Methods

The experiments were conducted on a static lead-bismuth experimental platform. The LBE (Pb 44.5 wt.–Bi 55.5 wt.%) was smelted from high-purity Pb (99.99%) and Bi (99.99%, Suzhou Chuanmao Metal Materials Co., Ltd., Suzhou, China) under an Ar atmosphere. During testing, Ar + 5% O_2_ (purity 99.999%, Air Liquide Tianjin Co., Ltd., Tianjin, China) was continuously introduced to maintain oxygen saturation, and the sensor insertion depth was approximately 20 mm. Stepwise heating and cooling tests were performed over the temperature range of 300–600 °C at a rate of 5 °C/min with a 50 °C interval. The accuracy tests involved holding at each temperature for over 5 h to ensure thermodynamic equilibrium. Short-term stability was evaluated by monitoring at 400 °C for 40 h, and long-term reliability was assessed over a cumulative operating time of more than 4000 h in a corrosion test loop (550–560 °C). The EMF was collected by a high-impedance electrometer (Yokogawa Electric Corporation, Tokyo, Japan; input impedance >200 TΩ) and corrected for the Seebeck thermoelectric potential *Uth* to obtain the experimental value E_exp_. Due to the complex fabrication process involving high-temperature brazing, YPSZ ceramic tube preparation, and precise reference electrode filling, one sensor was assembled and in-depth tested per condition group, following a single-variable comparison strategy.

### 2.3. Measurement Principle and Data Correction

In oxygen-saturated LBE, the theoretical EMF of the Bi/Bi_2_O_3_ reference electrode is given by the Nernst equation:

*Eth* (mV) = 119.8 − 0.0539 × *T* (K) (1)

The thermoelectric potential between the Mo wire and the 316L stainless steel setup was calculated and deducted as *Uth* = *C*(*T*_1_^2^ − *T*_2_^2^). This correction follows the classical method established by Schroer et al. [30], where the Seebeck effect between the dissimilar electrode lead materials (Mo wire and 316L stainless steel) superimposes a thermoelectric EMF onto the sensor’s intrinsic output [30]. The same correction approach has been widely adopted in domestic oxygen sensor research, as demonstrated by the work of Zhang [31], who provided a complete analysis of the Seebeck effect for the Mo/316L SS system and employed the identical correction formula for data processing.

### 2.4. Material Characterization and Simulation Methods

YPSZ powder was formed by dry pressing and sintering into discs with thicknesses of 1.1 mm and 1.5 mm (diameter 17 mm), and Pt paste was coated on both sides and fired integrally to form electrodes. EIS tests were performed using an Autolab PGSTAT302N electrochemical workstation (Metrohm Autolab B.V., Utrecht, The Netherlands) in a tube furnace in an air atmosphere over the temperature range of 200–600 °C, with data collected after holding at each 100 °C interval for 20 min (1 MHz–0.1 Hz). To ensure the analysis was grounded in established theory, the interpretation of the impedance data was based on the classical conduction theory of solid electrolytes [32,33]. A hierarchical equivalent circuit fitting was used to extract the bulk impedance *R_total_*; the ionic conductivity σ was calculated as *l*/(*R_total_* · *A*), and the oxygen ion migration activation energy was obtained using the Arrhenius equation.

Finite element simulations were performed using COMSOL Multiphysics^®^ 6.3 (COMSOL Inc., Stockholm, Sweden) with the Structural Mechanics Module. A two-dimensional axisymmetric model of the sensor was established, with a YPSZ tube outer diameter of 10 mm, a wall thickness of 1.5 mm, and a Bi filling height of 12 mm. The solid heat transfer, solid mechanics, and thermal expansion physics fields were coupled to simulate the transient stress distribution on the YPSZ tube caused by the solidification of Bi at 271 °C (volume expansion 3.3%) during cooling from 500 °C to room temperature. The YPSZ material parameters were set to those of 5 mol% Y_2_O_3_ stabilized zirconia: density 5950 kg/m^3^, thermal conductivity 3 W/(m·K), specific heat capacity 550 J/(kg·K), thermal expansion coefficient 8 × 10^−6^ K^−1^, Young’s modulus 2.05 × 10^11^ Pa, and Poisson’s ratio 0.222. For Bi, the solid-state parameters were density 9780 kg/m^3^, thermal conductivity 8 W/(m·K), specific heat capacity 120 J/(kg·K), thermal expansion coefficient 13.4 × 10^−6^ K^−1^, Young’s modulus 3.2 × 10^10^ Pa, and Poisson’s ratio 0.33; the liquid-state density and thermal conductivity were 10,050 kg/m^3^ and 15 W/(m·K), respectively. The boundary conditions were as follows: initial temperature 500 °C, external natural convection cooling (h = 10 W/(m^2^·K)), thermal contact resistance at the Bi/YPSZ interface (1 × 10^−5^ m^2^·K/W), and symmetry axis constraints.

## 3. Results and Discussion

### 3.1. Optimization of Reference Electrode Parameters

To investigate the effects of the Bi/Bi_2_O_3_ mass ratio and filling amount, four sensors were assembled with a fixed YPSZ wall thickness of 1.5 mm and Mo lead wire (Table 1).

#### 3.1.1. Activation Characteristics

**Figure 1:**
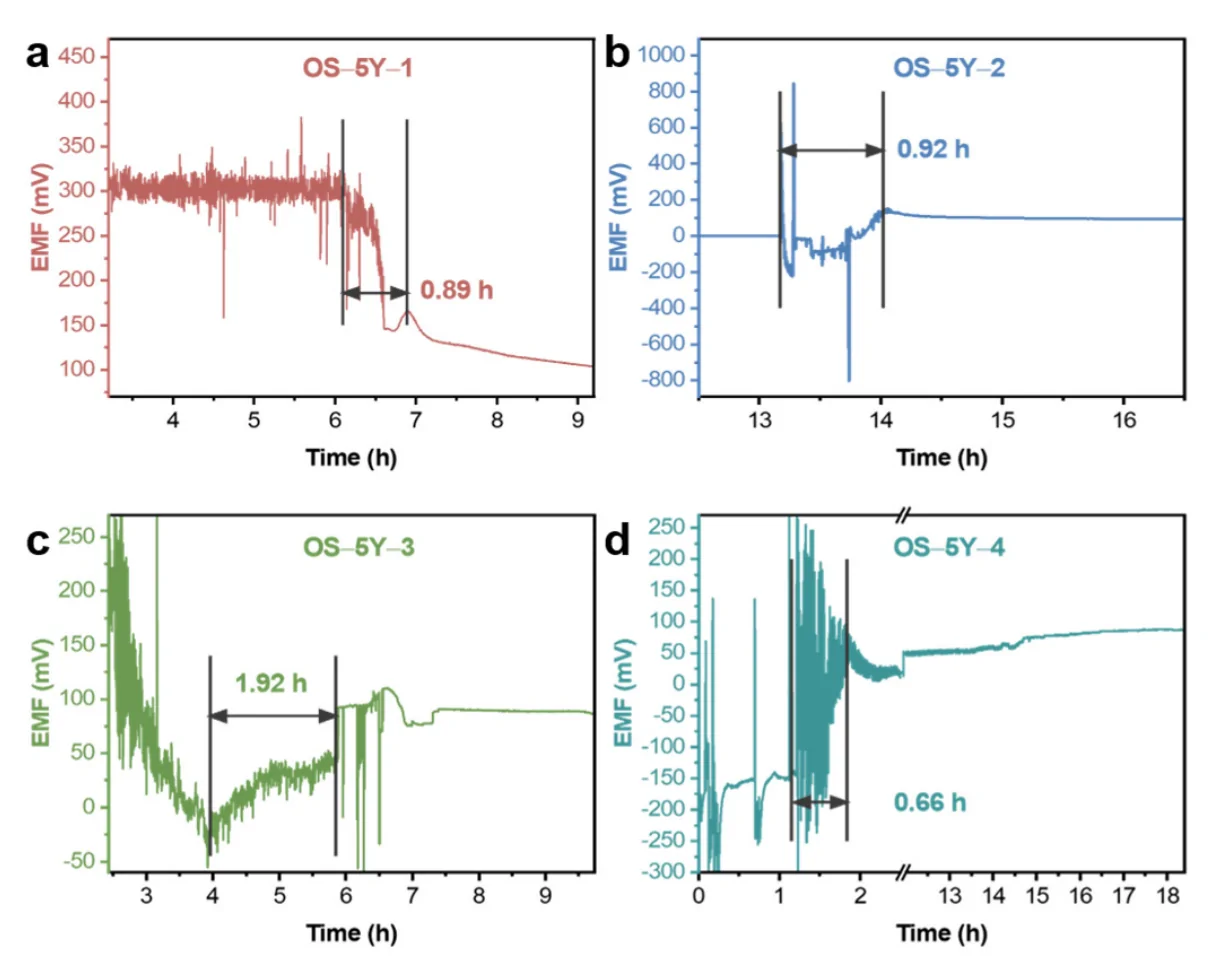
Activation curves of sensors with different Bi/Bi_2_O_3_ ratios and filling amounts in oxygen-saturated LBE at 400 °C: (**a**) OS-5Y-1 (90:10, 10 g); (**b**) OS-5Y-2 (95:5, 10 g); (**c**) OS-5Y-3 (95:5, 5.7 g); (**d**) OS-5Y-4 (95:5, 15 g).

The results of the in situ activation tests at 400 °C are shown in Figure 1. Under a fixed ratio of 95:5, OS-5Y-2 with 10 g had an activation time of 0.92 h and stabilized immediately after activation (Figure 1b); OS-5Y-3 with 5.7 g had an extended activation time of 1.92 h with severe fluctuations (Figure 1c); OS-5Y-4 with 15 g had the fastest activation (0.66 h), but the initial EMF was only about 50 mV, followed by a slow drift, approaching the theoretical value only after 13 h (Figure 1d). With a fixed filling amount of 10 g, the activation behaviors of the two ratios were similar (Figure 1a,b).

The above differences stem from the competition between the interfacial reaction area and the mass transport path. When the filling amount is insufficient, the effective contact area between Bi/Bi_2_O_3_ and YPSZ decreases, the number of three-phase boundary reaction sites is insufficient, the interfacial impedance increases, and the activation time is prolonged. When the filling amount is excessive, the excessively thick powder layer lengthens the diffusion path of oxygen ions within the reference electrode, causing a gradient distribution of oxygen potential within the thick layer. Consequently, the EMF requires a long time to converge. A filling amount of 10 g achieved the optimal balance between the number of reaction sites and the transport path, while the 95:5 ratio provided a stable two-phase oxygen partial pressure.

#### 3.1.2. Measurement Accuracy

**Figure 2:**
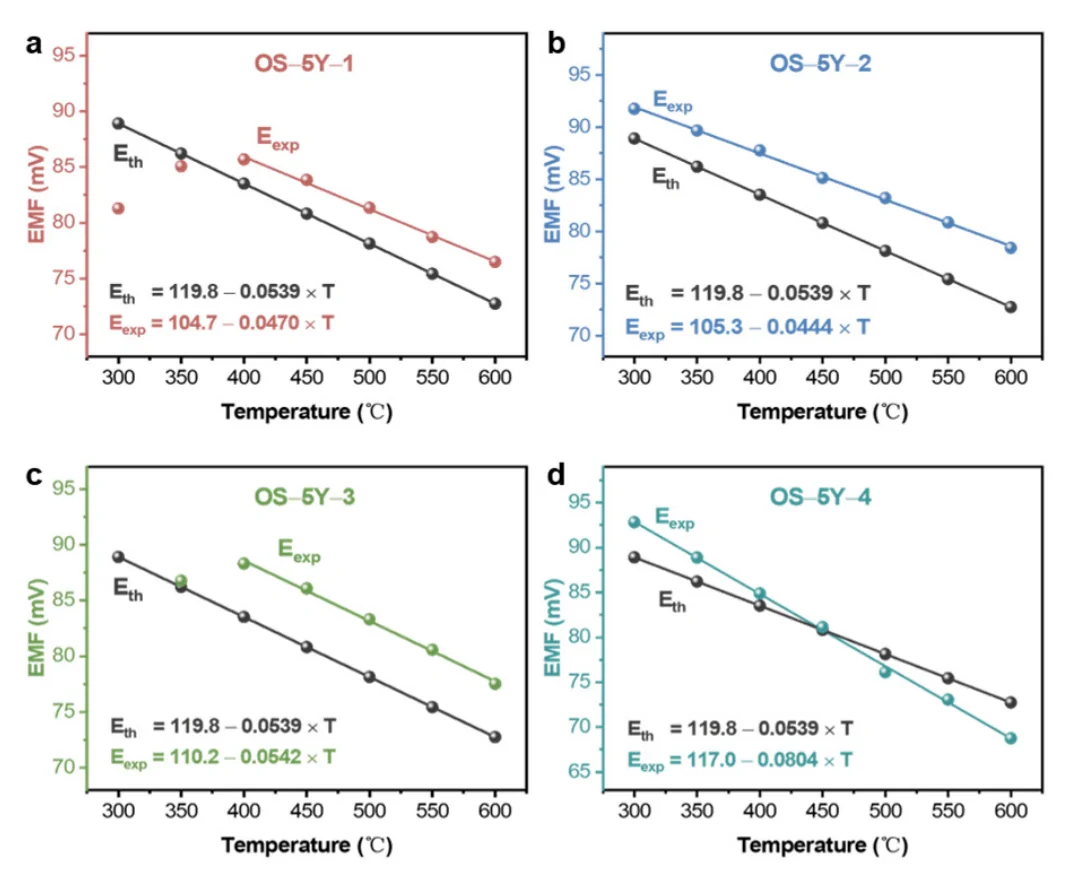
Comparison and linear fitting of experimental and theoretical EMF for sensors with different reference electrode parameters during the cooling stage from 300 to 600 °C: (**a**) OS-5Y-1; (**b**) OS-5Y-2; (**c**) OS-5Y-3; (**d**) OS-5Y-4.

The results of the stepwise cooling tests from 300 to 600 °C are shown in Figure 2 and Table 2. Consistent with previous studies [20], the data from the cooling stage, where the electrolyte was fully activated, are more reliable. OS-5Y-2 exhibited good linearity over the entire temperature range, with a fitting equation of E_exp_ = 105.3 − 0.0444 × T, a slope deviation of only 17.6%, and a relative error of only 3.13% at 300 °C. OS-5Y-1 and OS-5Y-3 showed an abnormal signal decrease below 350 °C, indicating low-temperature failure. This demonstrates that the excess Bi_2_O_3_ in the 90:10 ratio increased interface polarization at low temperatures, and the 5.7 g filling amount could not maintain a stable interface balance due to the insufficient total amount of active material. OS-5Y-4 showed no failure over the entire temperature range, with a relative error of only 0.38% at 400 °C; however, the slope deviation of the fitting equation E_exp_ = 117.0 − 0.0804 × T reached 49.2%, indicating a systematic deviation. The long drift during its activation phase originated from the slow homogenization of the oxygen potential within the thick powder layer during heating. The good single-point accuracy during the cooling phase was because the oxygen potential had basically equilibrated after prolonged high-temperature soaking, but the residual gradient within the thick layer varied nonlinearly with temperature, causing a systematic deviation in the output from the inherent slope of the Nernst equation. Its comprehensive performance was inferior to that of OS-5Y-2. A similar composition–oxygen potential correlation was also observed by Geng et al. [21] in their comparative study of Cu/Cu_2_O and Ni/NiO sensors, further confirming the decisive influence of the reference electrode composition on measurement accuracy.

The relative errors listed in Table 2 represent EMF errors. Upon converting the experimental EMF data to dissolved oxygen concentration using the Nernst equation, the corresponding oxygen concentration relative error for OS-5Y-2 was calculated to be 11.06% at 300 °C and 14.09% at 600 °C, with a full-range value of 11.06–14.31%. Since oxygen concentration and EMF are related through an exponential function (*C_O_* ∝ *exp*(−2*FE*/*RT*)), small EMF deviations are amplified when converted to oxygen concentration errors, resulting in higher percentage values. Considering that the oxygen control window in LBE typically spans several orders of magnitude (from 10^−8^ to 10^−6^ wt.%), an oxygen concentration error of <15% is fully acceptable for corrosion protection purposes.

Through a comprehensive comparison, a Bi/Bi_2_O_3_ mass ratio of 95:5 and a filling amount of 10 g were determined to be the optimal reference electrode formulation.

#### 3.1.3. Long-Term Service Performance

**Figure 3:**
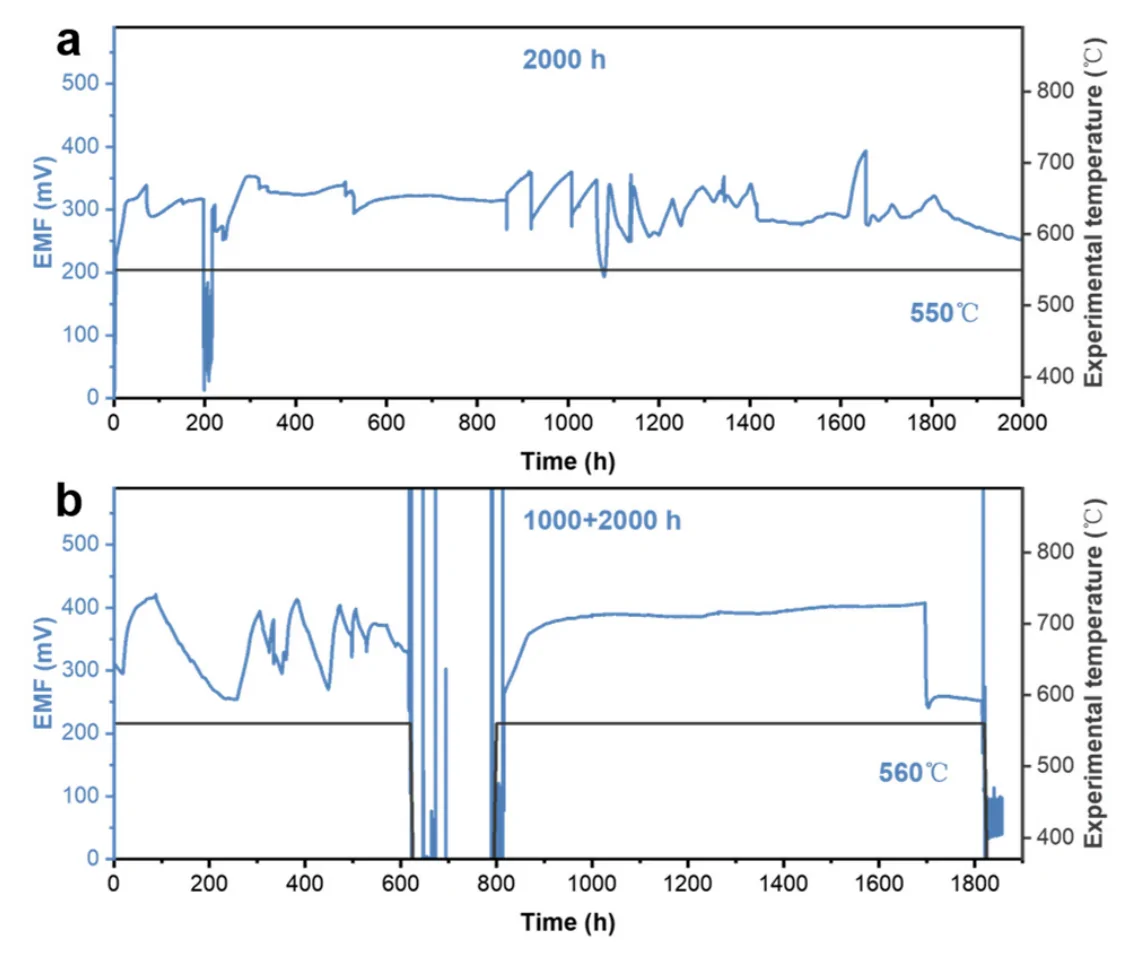
Long-term service EMF curves of the OS-5Y-2 sensor in the liquid lead-bismuth corrosion test loop: (**a**) 2000 h continuous test at 550 °C; (**b**) 1000 h + 1000 h staged test at 560 °C.

OS-5Y-2 was applied in a liquid lead-bismuth corrosion test loop for a cumulative operating time of over 4000 h (Figure 3). During the 2000 h continuous test at 550 °C, the EMF only fluctuated instantaneously when the atmosphere was switched and recovered immediately. In the staged 2000 h test at 560 °C, the sensor underwent shutdown-cooling-reactivation thermal cycles without any baseline drift in the signal. After the assessment, the YPSZ tube showed no cracking, and the seal remained intact, verifying the long-term reliability of the optimized formulation.

### 3.2. Influence of YPSZ Wall Thickness on Sensor Performance

Based on the determined optimal reference electrode (95:5, 10 g), the influence of the YPSZ wall thickness was further investigated. A 1.1 mm wall thickness YPSZ tube was introduced and matched with Mo and 316L stainless steel lead wires to rule out lead wire interference. The sensors involved are listed in Table 3.

#### 3.2.1. Activation Characteristics

**Figure 4:**
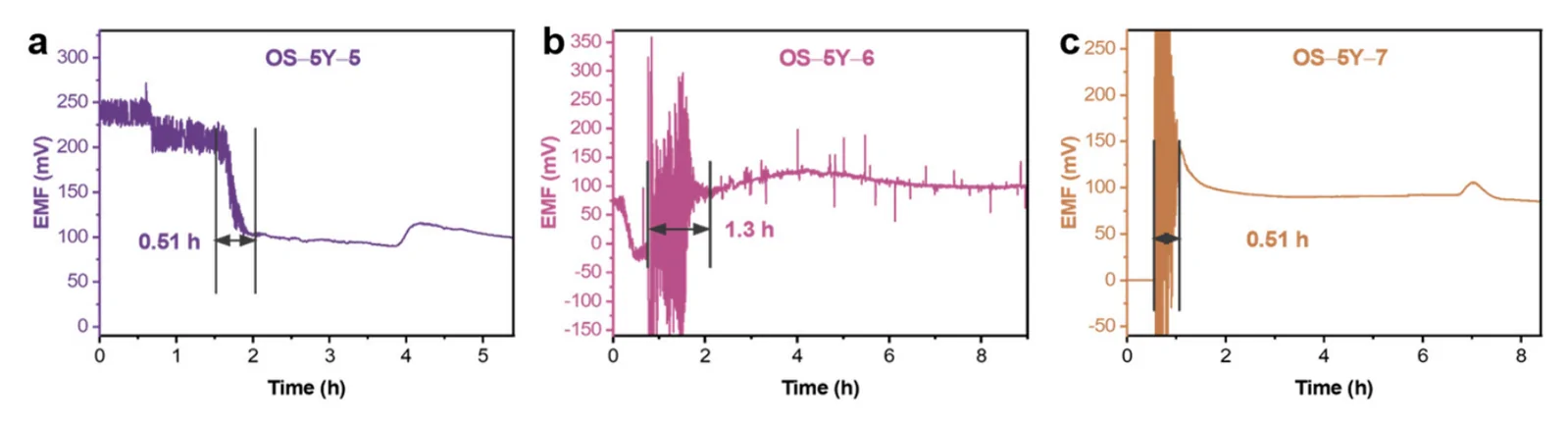
Activation curves of sensors with different wall thicknesses and lead wire materials at 400 °C: (**a**) OS-5Y-5 (1.1 mm, Mo); (**b**) OS-5Y-6 (1.5 mm, 316L SS); (**c**) OS-5Y-7 (1.1 mm, 316L SS).

The results of the in situ activation tests at 400 °C are shown in Figure 4. In the Mo wire system, the 1.5 mm thick-walled OS-5Y-2 had an activation time of 0.92 h with a smooth process and stabilized immediately after activation; the 1.1 mm thin-walled OS-5Y-5 completed activation in only 0.51 h, representing a 44.6% reduction, but high-frequency oscillations in the 100–250 mV range occurred during the initial activation phase and did not fully stabilize until 4 h later (Figure 4a). The 316L stainless steel wire system showed a consistent pattern: the 1.5 mm OS-5Y-6 had an activation time of 1.3 h, while the 1.1 mm OS-5Y-7 required only 0.51 h (a 60.8% reduction), but the latter also exhibited substantial jumps during the initial activation phase, with signal convergence significantly slower than that of the thick-walled sample (Figure 4b,c).

The consistent pattern in the two lead wire systems indicates that wall thickness is the intrinsic factor affecting the activation behavior. The advantage of the thin wall lies in shortening the transport path of oxygen ions across the electrolyte, reducing the bulk impedance, and, with a smaller thermal capacity and faster thermal response of the tube, allowing it to reach thermal equilibrium more quickly, thus leading to faster activation. However, the lower bulk impedance of the thin wall makes the interfacial electrochemical reaction more sensitive to minute fluctuations in oxygen concentration and temperature in the LBE, readily causing sustained oscillations before interfacial equilibrium is established. The higher bulk impedance of the thick wall acts as a buffer against electrochemical noise, resulting in a smoother signal change during the activation process, allowing it to enter a stable working state immediately after completion.

#### 3.2.2. Measurement Accuracy

**Figure 5:**
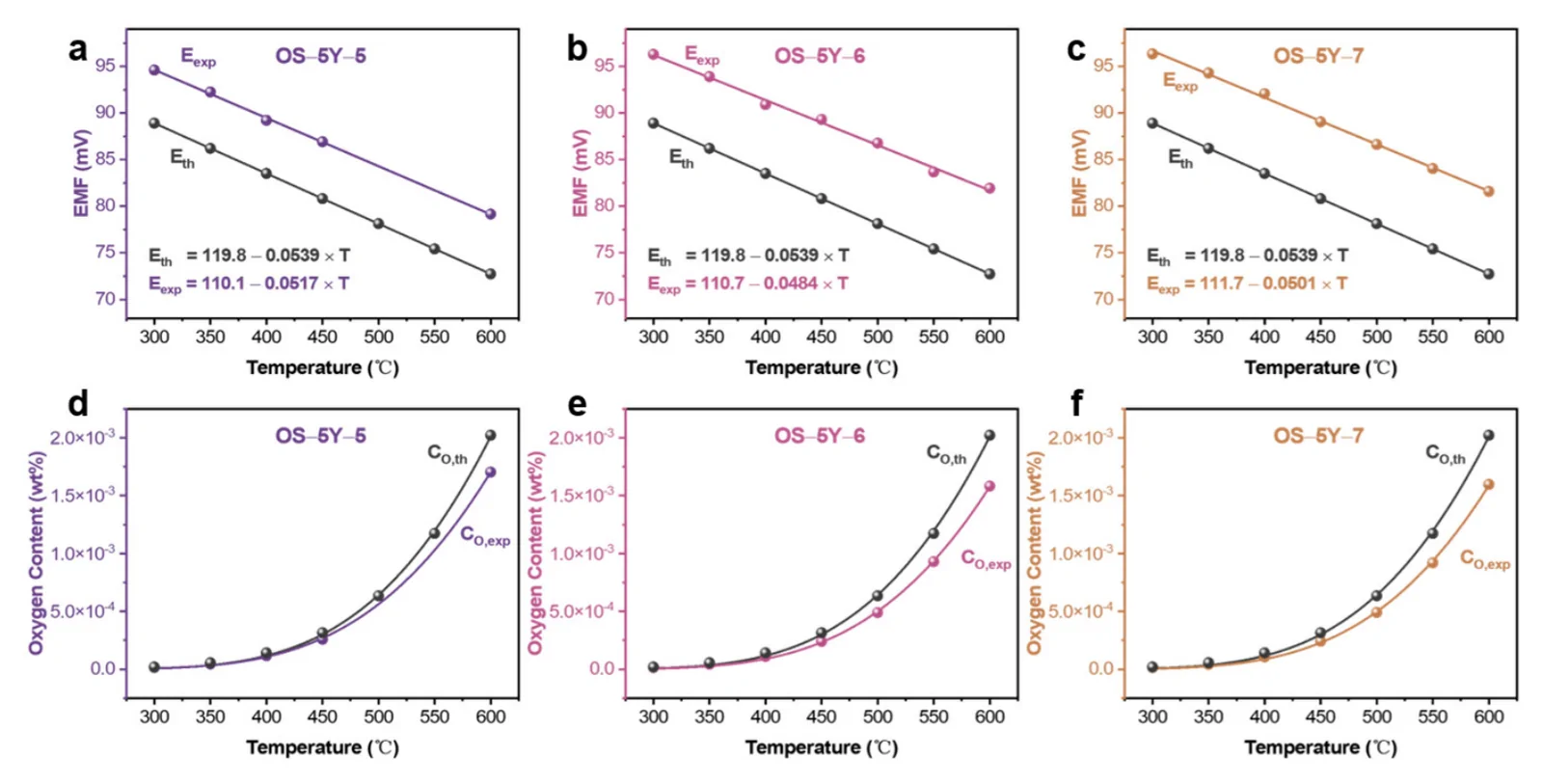
Measurement accuracy of sensors with different wall thicknesses and lead wire materials during the cooling stage from 300 to 600 °C: (**a**–**c**) comparison and linear fitting of experimental and theoretical EMF for (**a**) OS-5Y-5 (1.1 mm, Mo), (**b**) OS-5Y-6 (1.5 mm, 316L SS), and (**c**) OS-5Y-7 (1.1 mm, 316L SS); (**d**–**f**) comparison of the converted oxygen concentration from the corresponding sensor with the theoretical value for (**d**) OS-5Y-5, (**e**) OS-5Y-6, and (**f**) OS-5Y-7.

The stepwise cooling accuracy test results over 300–600 °C are shown in Figure 5 and Table 4. The EMF of all four sensors increased monotonically with decreasing temperature, consistent with the Nernst equation, and no low-temperature failure occurred for either wall thickness over the entire temperature range.

In the Mo wire system, the 1.5 mm OS-5Y-2 had a relative EMF error of only 3.13% at 300 °C, while the 1.1 mm OS-5Y-5 had a relative error of 6.41%, with the thick-walled sensor’s low-temperature accuracy being more than 50% better. In the stainless steel wire system, the error levels of OS-5Y-6 and OS-5Y-7 were similar at the same temperatures (8.30% and 8.36% at 300 °C, respectively), indicating that reducing the wall thickness did not sacrifice low-temperature accuracy. It should be noted that the errors for the stainless steel wire were generally higher than those for the Mo wire. This is because the Fe and Cr elements in 316L undergo a weak interface reaction with Bi/Bi_2_O_3_ at high temperatures, forming a high-impedance oxide layer [5,15]. This difference does not affect the judgment of the wall thickness effect—both lead wire systems confirmed that the thick-walled sensor has superior measurement accuracy under low-temperature steady-state conditions compared to the thin-walled one.

#### 3.2.3. Dynamic Response Characteristics

**Figure 6:**
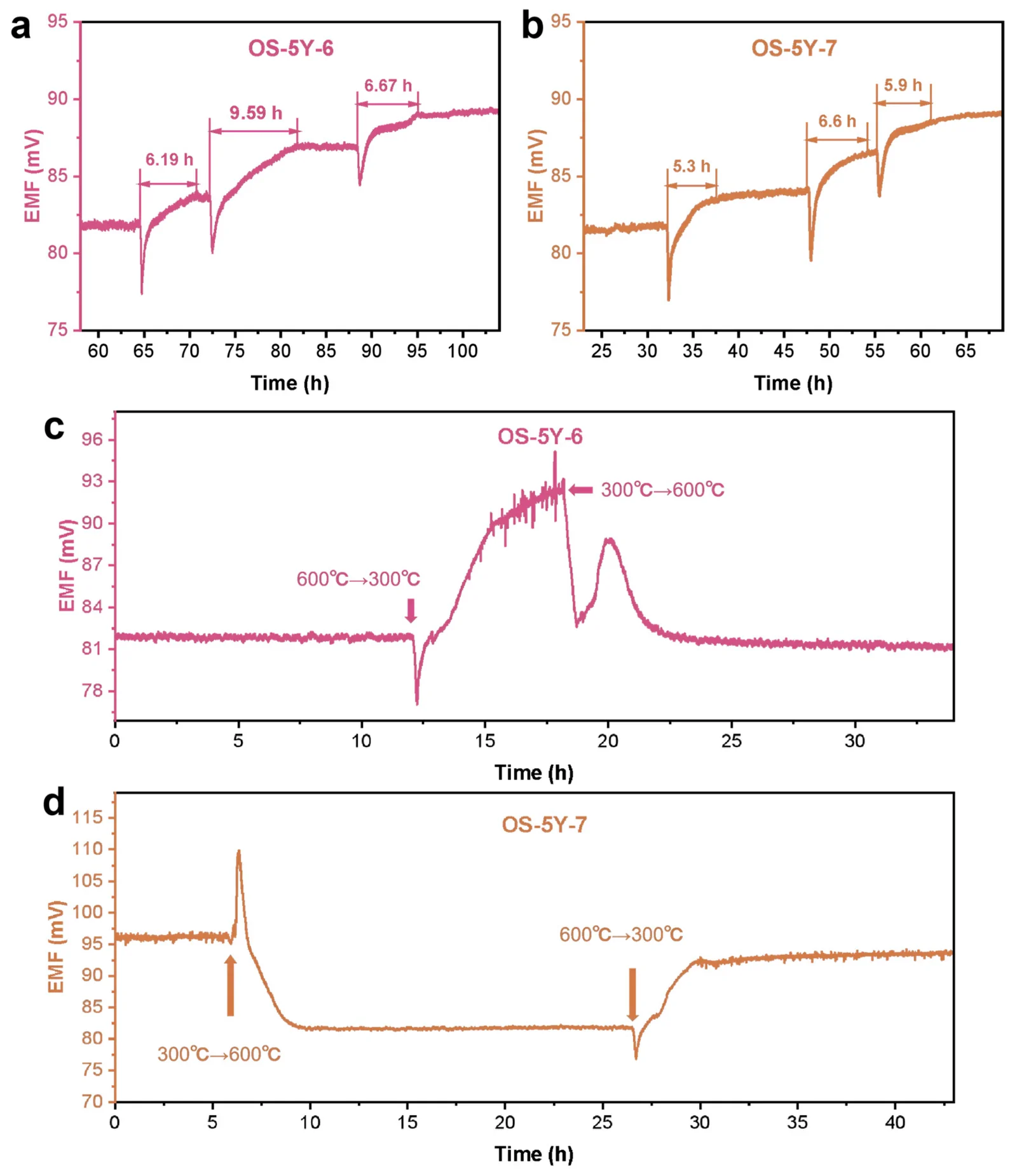
EMF response curves of sensors with different wall thicknesses under stepwise cooling and temperature step changes: (**a**) OS-5Y-6 (1.5 mm) stepwise cooling 600 → 550 → 500 → 450 °C; (**b**) OS-5Y-7 (1.1 mm) stepwise cooling; (**c**) OS-5Y-6 cooling step 600 → 300 °C; (**d**) OS-5Y-7 heating step 300 → 600 °C.

The influence of wall thickness on the dynamic response was investigated using OS-5Y-6 and OS-5Y-7 from the 316L stainless steel wire system, with the results shown in Figure 6. During stepwise cooling, the effective response time of the 1.1 mm OS-5Y-7 at each temperature step was shorter than that of the 1.5 mm OS-5Y-6, with an average reduction of about 19%, and the largest acceleration (31.2%) was observed in the 550 → 500 °C interval. In the temperature step experiment, OS-5Y-7 quickly converged to a stable value after heating from 300 to 600 °C without obvious oscillations; OS-5Y-6, on the other hand, exhibited high-frequency oscillations lasting over 5 h, with a significant response lag. The faster response and smoother signal of the thin-walled sensor were also observed in the cooling step from 600 to 300 °C.

The difference in response speed between the two wall thicknesses originates from the kinetics of oxygen ion transport. When the temperature changes, the EMF response of the sensor is jointly determined by the transport rate of oxygen ions across the electrolyte and the rate at which the interfacial oxygen concentration difference reaches equilibrium. The thin-walled electrolyte shortens the transport path and reduces the bulk impedance, allowing oxygen ions to complete the transmembrane migration more quickly and rapidly establish a steady-state oxygen concentration difference at the new temperature, hence the faster response and smaller oscillations. As the temperature decreases, the conductivity of YPSZ decreases exponentially, and the influence of wall thickness on the transport rate becomes more significant, making the response advantage of the thin wall particularly prominent in the high-to-low temperature transition range.

Considering the three aspects of performance, the wall thickness exhibits a bidirectional regulating effect on the sensor: the 1.1 mm thin wall offers fast response and rapid activation, making it suitable for scenarios requiring quick start-up and frequent operating condition fluctuations; the 1.5 mm thick wall provides higher low-temperature measurement accuracy, a smoother signal, and, with its larger wall cross-section, stronger resistance to thermal shock and cracking. Given that oxygen monitoring in lead-based reactors primarily involves long-term steady-state operation, where the requirements for accuracy and stability take precedence over response speed, and in view of the excellent long-term reliability demonstrated by OS-5Y-2 in the 4000 h assessment, the 1.5 mm YPSZ wall thickness was selected as the optimal solution.

### 3.3. Material Characteristics and Mechanism Analysis

#### 3.3.1. Regulation of Ion Conduction by YPSZ Wall Thickness—EIS Analysis

**Figure 7:**
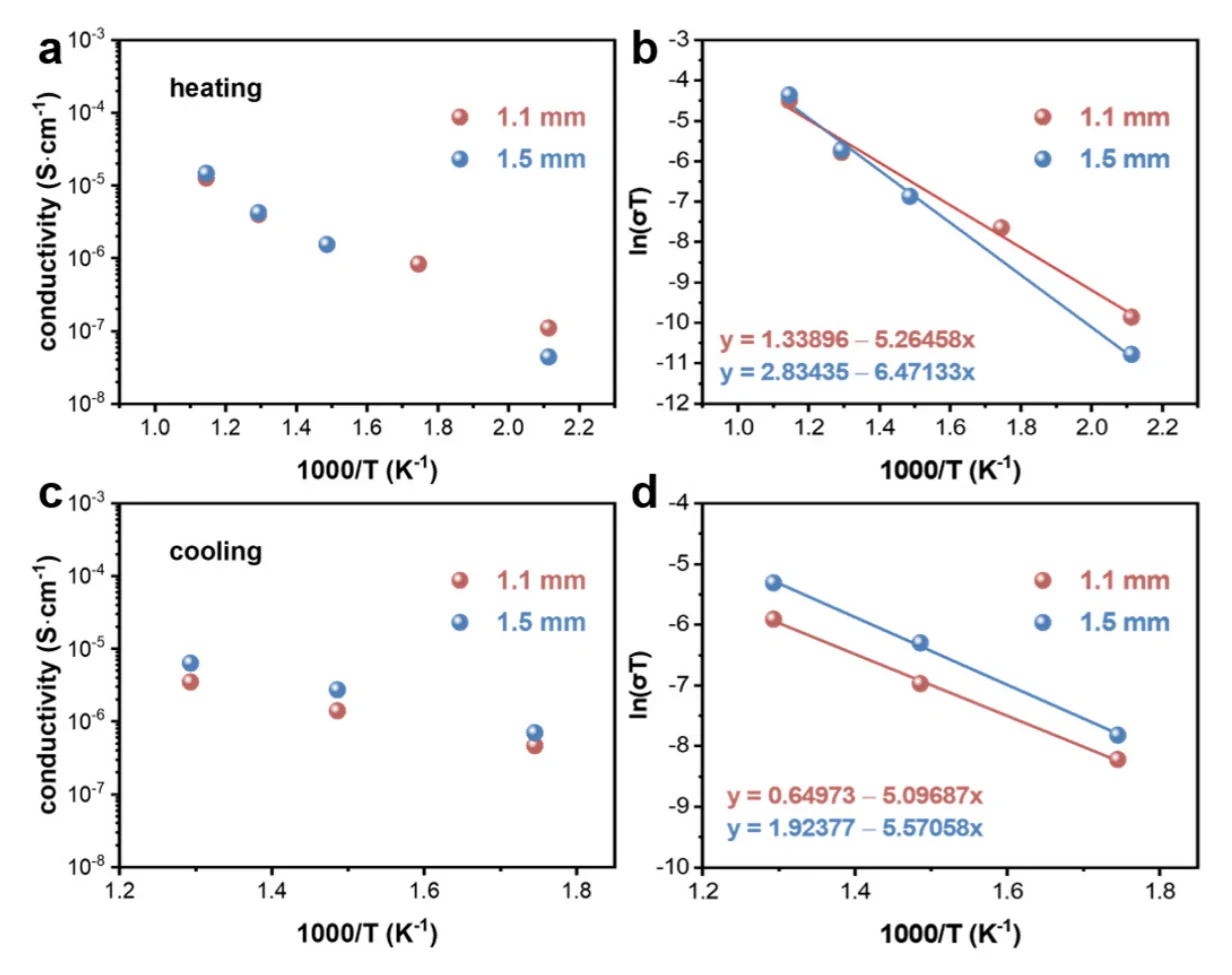
Arrhenius fitting curves of ionic conductivity for YPSZ electrolytes with different thicknesses during heating and cooling between 200 and 600 °C.

To fundamentally reveal the reason for the superior low-temperature accuracy of the thick-walled sensor described in Section 3.2, EIS tests were conducted on YPSZ samples of 1.1 mm and 1.5 mm thicknesses during heating and cooling between 200 and 600 °C. Based on the classical conduction theory of solid electrolytes [32,33], a hierarchical equivalent circuit fitting was used to extract the bulk impedance *R_total_*. The ionic conductivity σ was calculated as *l*/(*R_total_* · *A*), and the oxygen ion migration activation energy was obtained using the Arrhenius equation. The results are summarized in Table 5 and Figure 7.

During the heating process, the impedance of both electrolyte thicknesses decreased exponentially with increasing temperature. The 1.1 mm sample formed an effective conduction pathway at 300 °C (σ = 8.33 × 10^−7^ S/cm), while the 1.5 mm sample was still in an unactivated state, indicating an activation temperature difference of approximately 100 °C. Arrhenius fitting yielded an activation energy of 0.45 eV for the thin wall and 0.56 eV for the thick wall (Table 6). The lower migration energy barrier of the thin wall originates from its shorter conduction path, which weakens the cumulative grain boundary blocking effect, consistent with the macroscopic observation of faster activation in thin-walled sensors described in Section 3.2.1.

During the cooling process, the impedance trend for the two thicknesses was significantly reversed: at the same temperature, the bulk impedance of the 1.5 mm thick wall was lower than that of the 1.1 mm thin wall. At 300 °C, the thick wall impedance was only 66.7% of the thin wall (200,020 Ω vs. 299,980 Ω), and at 400 °C, it was 69.9% (69,970 Ω vs. 100,030 Ω). Arrhenius fitting revealed the origin of this reversal: the activation energy of the thick wall decreased from 0.56 eV in the heating stage to 0.48 eV after cooling (a reduction of 14.3%), while the thin wall only decreased from 0.45 eV to 0.44 eV (a reduction of 2.2%).

The physical nature of this “thermal hysteresis” effect is as follows: the thick-walled YPSZ inherently possesses more grain boundary defects and residual sintering stress. The high-temperature soak at 600 °C serves as an in situ annealing process, where grain boundaries fully relax, and the distribution of oxygen vacancies becomes more uniform, thereby significantly lowering the migration energy barrier. Concurrently, the larger thermal capacity of the thick wall results in a smaller internal temperature gradient during cooling. This avoids local lattice distortion and partial closure of oxygen ion pathways that can be caused by rapid cooling in the thin wall, allowing the optimized conduction network formed at high temperatures to be fully retained. In contrast, the thin wall has fewer initial defects, leaving limited room for improvement at high temperatures. Furthermore, rapid cooling can lead to pathway closure, resulting in only a minimal decrease in activation energy.

This microscopic mechanism directly explains the macroscopic performance difference observed in Section 3.2.2: The lower bulk impedance of the thick-walled electrolyte during the cooling stage reduces the total internal resistance of the sensor, leading to higher oxygen ion conduction efficiency and a more stable interfacial oxygen concentration difference equilibrium. Consequently, the EMF output is closer to the Nernst theoretical value. This accounts for why the relative error at 300 °C for the 1.5 mm thick-walled OS-5Y-2 (3.13%) was more than 50% lower than that of the 1.1 mm thin-walled OS-5Y-5 (6.41%). The low-temperature conductivity advantage gained by the thick-walled YPSZ after sufficient high-temperature activation provides the intrinsic physical basis for the sensor’s wide-temperature-range and high-accuracy performance.

#### 3.3.2. Bi Solidification Phase Transformation Stress and Electrolyte Cracking—COMSOL Simulation

**Figure 8:**
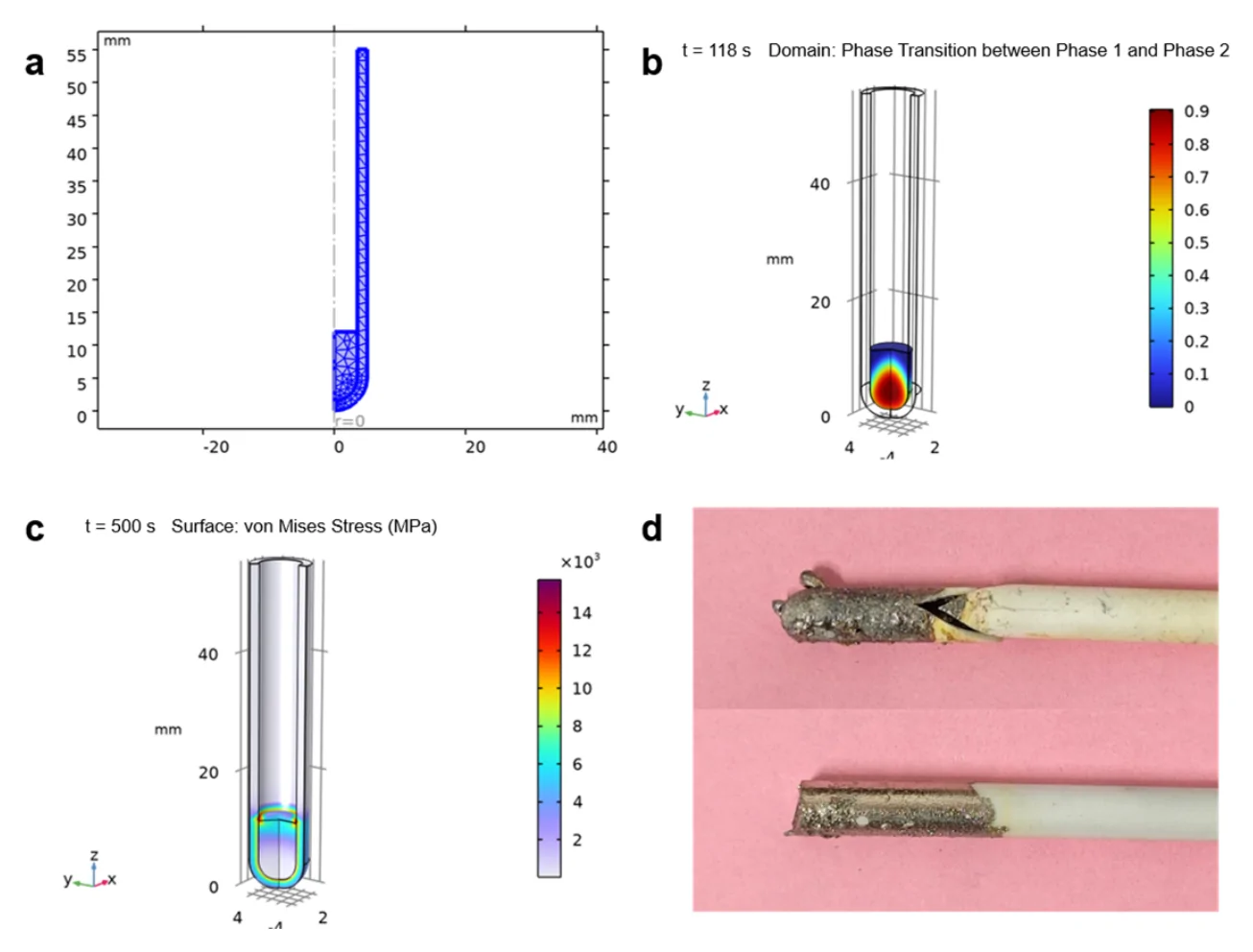
COMSOL Multiphysics simulation results of YPSZ electrolyte cracking induced by Bi solidification phase transformation: (**a**) 2D axisymmetric finite element model of the sensor; (**b**) liquid–solid phase transformation distribution of the Bi electrode at 118 s of cooling; (**c**) von Mises equivalent stress distribution cloud map of the YPSZ tube at room temperature; (**d**) cracking site of an experimentally failed sensor.

In addition to electrochemical performance, the structural reliability of the sensor under repeated thermal cycles is equally critical. In the experiments, some sensors experienced YPSZ tube cracking after multiple start–stop cycles. To reveal the mechanical mechanism, a two-dimensional axisymmetric finite element model was established (Figure 8a). The simulation modeled the process of cooling from 500 °C to room temperature, during which Bi solidifies at 271 °C, accompanied by a 3.3% volume expansion.

The simulation results show that the solidification of Bi begins from the bottom and outer side of the tube, with the solid–liquid interface gradually advancing toward the axis and the top (Figure 8b). The von Mises equivalent stress distribution at room temperature (Figure 8c) indicates that the maximum stress is concentrated at the top inner interface edge of the Bi filling section. This location is a point of abrupt structural constraint—the lower part is subjected to axial compression from the expanding solidified Bi, while the upper part is a free cavity without filling. The expansion stress cannot be released along the axial direction and acts entirely perpendicularly on the YPSZ inner wall at the contact edge, forming a strong stress concentration and becoming the primary danger zone for crack initiation.

The crack initiation site predicted by the simulation is in full agreement with the cracking origin of the experimentally failed sensor (Figure 8d). The cracks in the failed sensors all originated at the top of the contact interface between the Bi electrode and the YPSZ tube. Further analysis indicates that when the Bi filling is uniform, the top is free of sharp corners, and the YPSZ is free of initial microcracks, the stress concentration can be effectively mitigated. Conversely, uneven filling or the presence of sharp corners at the interface will cause local stress to far exceed the fracture strength of the ceramic, leading to early cracking. This explains why OS-5Y-2 remained intact after multiple thermal cycles and over 4000 h of assessment—in addition to the optimization of electrochemical performance, a strict assembly process is also key to ensuring long-term reliability.

The current simulation employed a linear elastic constitutive model and did not account for residual stresses from brazing at the ceramic–metal seal. Brazing may introduce additional tensile stresses in the YPSZ tube; however, these were typically localized at the seal region, far from the top of the Bi filling section, where cracks initiated. While the combined effect could slightly modify the absolute stress magnitude, it would not change the identified crack initiation site. Future modeling efforts will incorporate the full thermal history of the sensor.

## 4. Conclusions

(1) A reference electrode with a Bi/Bi_2_O_3_ mass ratio of 95:5 and a filling amount of 10 g can maintain a stable two-phase equilibrium oxygen potential across the entire temperature range of 300–600 °C. The sensor assembled based on this formulation successfully extended the lower stable operating limit from 350 °C (typical for this type) to 300 °C, achieving a relative EMF error of only 3.13% at 300 °C. It operated for over 4000 h cumulatively and experienced multiple thermal cycles without signal drift or electrolyte cracking.
(2) The YPSZ wall thickness has a bidirectional regulating effect on sensor performance. The 1.5 mm thick wall, after high-temperature activation at 600 °C, exhibited a decrease in its oxygen ion migration activation energy to 0.48 eV during the cooling process. Its bulk impedance at 300 °C was only 66.7% of that of the 1.1 mm thin wall, and its low-temperature steady-state measurement accuracy improved by over 50% compared to the thin wall. The 1.1 mm thin wall reduced activation time by over 44% and accelerated the temperature step response by about 19% to 31%, making it suitable for dynamic operating conditions.
(3) EIS analysis revealed the intrinsic mechanism for the high accuracy of the thick wall at low temperatures: The thick wall has more initial grain boundary defects, which are repaired to a greater extent during high-temperature activation. Its larger thermal capacity also ensures a uniform temperature field during cooling, allowing the optimized conduction network from high temperatures to be fully retained. COMSOL simulations precisely located the stress concentration zone induced by the Bi solidification phase transformation (3.3% volume expansion) at the top inner interface edge of the filling section, consistent with the experimental crack initiation site. This confirms the critical role of filling uniformity in suppressing cracking.
(4) Although the optimized sensor has demonstrated reliable operation down to 300 °C and over 4000 h of long-term stability, further investigation is required for its deployment under actual reactor conditions. Dedicated tests under controlled low-oxygen partial pressures—closer to actual reactor conditions—are planned to fully characterize the dynamic performance of the sensor. The stability of the sensor under neutron/gamma irradiation also needs to be evaluated, as irradiation may influence oxygen vacancy mobility in YPSZ and affect long-term signal reliability. Furthermore, based on the stress concentration zones identified by the COMSOL simulation, further optimization of the filling geometry and packing process should be pursued to further enhance the structural reliability of the sensor under repeated thermal cycling.

## Figures and Tables

**Table 1:** Sensor parameters for the reference electrode optimization experiment.

Sensor ID	Bi:Bi_2_O_3_	Filling Amount/g	YPSZ Wall Thickness/mm	Lead Wire
OS-5Y-1	90:10	10	1.5	Mo
OS-5Y-2	95:5	10	1.5	Mo
OS-5Y-3	95:5	5.7	1.5	Mo
OS-5Y-4	95:5	15	1.5	Mo

**Table 2:** EMF errors of sensors with different reference electrode parameters during the cooling stage.

Sensor	300 °C	400 °C	500 °C	600 °C
OS-5Y-1	7.62 (8.57%)	2.16 (2.59%)	3.22 (4.12%)	3.75 (5.15%)
OS-5Y-2	2.79 (3.13%)	4.25 (5.09%)	5.07 (6.49%)	5.68 (7.81%)
OS-5Y-3	---	4.79 (5.74%)	5.18 (6.63%)	4.78 (6.57%)
OS-5Y-4	3.91 (4.39%)	1.36 (1.63%)	2.02 (2.58%)	3.99 (5.48%)

Note: Data are absolute errors (mV), with relative errors in parentheses. OS-5Y-3 was not tested at 300 °C and 350 °C.

**Table 3:** Sensor parameters for the wall thickness comparison experiment.

Sensor ID	Bi:Bi_2_O_3_	Filling Amount/g	YPSZ Wall Thickness/mm	Lead Wire Material
OS-5Y-2	95:5	10	1.5	Mo
OS-5Y-5	95:5	10	1.1	Mo
OS-5Y-6	95:5	10	1.5	316L SS
OS-5Y-7	95:5	10	1.1	316L SS

**Table 4:** Summary of EMF errors during the cooling stage for sensors with different wall thicknesses and lead wire materials.

Temperature/°C	OS-5Y-2 (1.5 mm, Mo)	OS-5Y-5 (1.1 mm, Mo)	OS-5Y-6 (1.5 mm, 316L)	OS-5Y-7 (1.1 mm, 316L)
300	2.79 (3.13%)	5.70 (6.41%)	7.38 (8.30%)	7.43 (8.36%)
400	4.25 (5.09%)	5.69 (6.81%)	7.39 (8.85%)	8.55 (10.24%)
500	5.07 (6.49%)	---	8.64 (11.06%)	8.50 (10.88%)
600	5.68 (7.81%)	6.42 (8.83%)	9.19 (12.63%)	8.85 (12.17%)

Note: Data are absolute errors (mV), with relative errors in parentheses. OS-5Y-5 was not tested at 500 °C and 550 °C.

**Table 5:** Ionic conductivity and Arrhenius parameters of YPSZ electrolytes with different thicknesses.

Thickness/mm	Thermal History	Temperature/°C	R_total_/Ω	σ/(S·cm^−1^)	ln(σT)
1.1	Heating	200	1,269,300	1.10 × 10^−7^	−9.86
1.1	Heating	300	167,960	8.33 × 10^−7^	−7.65
1.1	Heating	500	35,020	4.00 × 10^−6^	−5.78
1.1	Heating	600	11,020	1.27 × 10^−5^	−4.50
1.1	Cooling	300	299,980	4.67 × 10^−7^	−8.22
1.1	Cooling	400	100,030	1.40 × 10^−6^	−6.97
1.1	Cooling	500	40,010	3.50 × 10^−6^	−5.91
1.5	Heating	200	4,337,400	4.41 × 10^−8^	−10.78
1.5	Heating	400	123,646	1.55 × 10^−6^	−6.87
1.5	Heating	500	44,980	4.25 × 10^−6^	−5.72
1.5	Heating	600	12,980	1.47 × 10^−5^	−4.36
1.5	Cooling	300	200,020	7.00 × 10^−7^	−7.82
1.5	Cooling	400	69,970	2.73 × 10^−6^	−6.30
1.5	Cooling	500	30,020	6.37 × 10^−6^	−5.31

**Table 6:** Fitted results of oxygen ion migration activation energy for YPSZ electrolytes with different thicknesses.

Electrolyte Thickness	Test Condition	Fitting Slope	Activation Energy Ea/eV
1.1 mm	Heating	−5.2646	0.45
1.1 mm	Cooling	−5.0949	0.44
1.5 mm	Heating	−6.4713	0.56
1.5 mm	Cooling	−5.5706	0.48

## Data Availability

The original contributions presented in this study are included in the article. Further inquiries can be directed to the corresponding author.

## References

[1] Yang Q., Zheng Y., Wu H. (2025). Advancements and Challenges in Small Modular Lead/Lead Bismuth Eutectic Cooled Fast Reactors: A 30-Year Overview. Ann. Nucl. Energy.

[2] Wu Y. (2016). Design and R&D Progress of China Lead-Based Reactor for ADS Research Facility. Engineering.

[3] Gavrea R.C., Surducan E., Hirian R., Zagrai M., Rednic V. (2025). Structural, Thermophysical, and Radiation Shielding Properties of Lead-Bismuth Eutectic (LBE) Synthesized by Induction Melting. Crystals.

[4] Gong X., Short M.P., Auger T., Charalampopoulou E., Lambrinou K. (2022). Environmental Degradation of Structural Materials in Liquid Lead- and Lead-Bismuth Eutectic-Cooled Reactors. Prog. Mater. Sci..

[5] Wang W., Yang C., You Y., Yin H. (2023). A Review of Corrosion Behavior of Structural Steel in Liquid Lead-Bismuth Eutectic. Crystals.

[6] Li N. (2002). Active Control of Oxygen in Molten Lead-Bismuth Eutectic Systems to Prevent Steel Corrosion and Coolant Contamination. J. Nucl. Mater..

[7] Marino A., Lim J., Aerts A. (2017). Active Oxygen Control by a PbO Mass Exchanger in the Liquid Lead-Bismuth Eutectic Loop: MEXICO. J. Nucl. Sci. Technol..

[8] Qin B., Lu S.H., Liu S.H., Zhang J., Long B. (2025). Research Progress on Oxygen Measurement and Control in Lead-Bismuth Alloys for Material Corrosion Protection. Mater. Rep..

[9] He M.Y., Kang H.J., Lu S.T., Yao Z., Qin W., Wu X. (2023). Challenges and Solutions to the Application of Lead-Bismuth Eutectic Alloy Coolant. Rare Met. Mater. Eng..

[10] Liu C.F., Chen K., Ge F.F., Wang R., Huang Q. (2025). Research Progress on Lead-Bismuth Corrosion Resistant Coatings for Nuclear Applications. J. Inorg. Mater..

[11] Tan J.B., Zhang X.R., Xue B.Q., Zhang Z.Y., Wu X.Q. (2025). Research Progress on Environmental Compatibility Evaluation of Stainless Steels in Liquid Lead-Bismuth for Lead-Cooled Fast Reactors. Nucl. Tech..

[12] Orlov S.N., Bogachev N.A., Mereshchenko A.S., Zmitrodan A.A., Skripkin M.Y. (2023). Electrochemical Sensors for Controlling Oxygen Content and Corrosion Processes in Lead-Bismuth Eutectic Coolant—State of the Art. Sensors.

[13] Shooshtari M., Salehi A. (2022). An Electronic Nose Based on Carbon Nanotube-Titanium Dioxide Hybrid Nanostructures for Detection and Discrimination of Volatile Organic Compounds. Sens. Actuators B Chem..

[14] Li H., Zhu H.P., Zhu Y.Q., Wu W., Liang R., Wu H., Liu F., Niu F. (2025). Performance of Potentiometric Oxygen Sensors with LSM Composite Electrode in Saturated Oxygen Liquid LBE. Ann. Nucl. Energy.

[15] Schroer C., Wedemeyer O., Konys J. (2011). Aspects of Minimizing Steel Corrosion in Liquid Lead-Alloys by Addition of Oxygen. Nucl. Eng. Des..

[16] Lim J., Marien A., Rosseel K., Aerts A., Bosch J.V.D. (2012). Accuracy of Potentiometric Oxygen Sensors with Bi/Bi_2_O_3_ Reference Electrode for Use in Liquid LBE. J. Nucl. Mater..

[17] Lim J., Manfredi G., Marien A., Bosch J.V.D. (2013). Performance of Potentiometric Oxygen Sensors with LSM-GDC Composite Electrode in Liquid LBE at Low Temperatures. Sens. Actuators B Chem..

[18] Bassini S., Antonelli A., Di Piazza I., Tarantino M. (2017). Oxygen Sensors for Heavy Liquid Metal Coolants: Calibration and Assessment of the Minimum Reading Temperature. J. Nucl. Mater..

[19] Qin B., Fu X.G., Ma H.R., Zhang J.Q., Ren L.X., Long B. (2019). Preliminary Experimental Study on Gas-Phase Oxygen Content Control in Lead-Bismuth Alloys. Mater. Rep..

[20] Tu X., Zhu H.P., Wu W.H., Wang X., Guo Z., Wang T., Li W., Yang L., Liang R., Li H. (2024). Influence of Solid Electrolyte Material Selection on Electromotive Force Characteristics of Oxygen Sensor for Lead-Cooled Fast Reactor. Prog. Nucl. Energy.

[21] Geng T.J., Sheng Z.H., Wang S.F., Lyu H., Liu F. (2026). Performance Evaluation of Cu/Cu_2_O and Ni/NiO Based Potentiometric Oxygen Sensors in Saturated Oxygen Liquid Lead-Bismuth Eutectic. Nucl. Eng. Des..

[22] Guo Z.P., Wu W., Zhu H., Wang T., Li W., Liang R., Li H., Yang L., Sheng Z., Liu F. (2025). Temperature Effect on the Oxygen Control Efficiency of LSCF-Type Oxygen Pump for LBE. Prog. Nucl. Energy.

[23] Shooshtari M. (2025). Gold-Decorated Vertically Aligned Carbon Nanofibers for High-Performance Room-Temperature Ethanol Sensing. Microchim. Acta.

[24] Wang Y.Q., Huang Q.Y., Wu X., Wu B., Zhang M., Gao S. (2015). Accuracy and Stability Testing of Bi/Bi_2_O_3_ Oxygen Sensors in Lead-Bismuth Alloy. At. Energy Sci. Technol..

[25] Zhu H.P., Zhang Y., Zhao Y.G., Liu X.D., Chang B.C., Li X.B., Niu F.L., Ma Y., Zhao P. (2020). Performance Study of Cu/Cu_2_O Reference Electrode LBE Oxygen Sensor. Nucl. Tech..

[26] Li S.X. (2024). Performance Study of Low-Temperature Oxygen Sensors in Saturated Oxygen Concentration Lead-Bismuth Alloy. Master’s Thesis.

[27] Marien A., Lim J., Rosseel K., Vandermeulen W., Bosch J.V.D. (2012). Solid Electrolytes for Use in Lead-Bismuth Eutectic Cooled Nuclear Reactors. J. Nucl. Mater..

[28] Xu Y.H., Niu F.L., Zhang Y., Zhao Y.G. (2020). Performance Testing and Research on Bi/Bi_2_O_3_ Oxygen Sensor in Saturated Oxygen Concentration Lead-Bismuth Alloy. Nucl. Power Eng..

[29] Wang L., Shi J.Y., Chang H.J., Qin T. (2024). Design and Optimization of Oxygen Probe for Liquid Lead-Bismuth Alloy. Nucl. Saf..

[30] Schroer C., Konys J., Verdager A., Abellà J., Gessi A., Kobzova A., Babayan S., Courouau J.-L. (2011). Design and Testing of Electrochemical Oxygen Sensors for Service in Liquid Lead Alloys. J. Nucl. Mater..

[31] Zhang Y. (2019). Research and Development of Oxygen Concentration Measuring Instrument for Liquid Lead-Bismuth Alloy and Its Performance. Master’s Thesis.

[32] Subbarao E.C. (1980). Solid Electrolytes and Their Applications.

[33] Wang C.Z. (2000). Solid Electrolytes and Chemical Sensors.

